# Perinatal Depression and Anxiety Symptoms, Parental Bonding and Dyadic Sensitivity in Mother–Baby Interactions at Three Months Post-Partum

**DOI:** 10.3390/ijerph20054253

**Published:** 2023-02-27

**Authors:** Anna Maria Della Vedova, Fabrizio Santoniccolo, Cristina Sechi, Tommaso Trombetta

**Affiliations:** 1 Department of Clinical and Experimental Sciences, University of Brescia, Viale Europa 11, 25123 Brescia, Italy; 2 Department of Psychology, University of Turin, Via Verdi 10, 10124 Torino, Italy; 3 Department of Pedagogy, Psychology and Philosophy, University of Cagliari, Via Is Mirrionis 1, 09126 Cagliari, Italy

**Keywords:** sensitivity, attachment, perinatal depression, temperament, parental bonding

## Abstract

The quality of the early parent–infant relationship is crucial for the child’s optimal development, and parental sensitivity plays a key role in early interactions. The purpose of the study was to evaluate the influence of maternal perinatal depression and anxiety symptoms on dyadic sensitivity at three months post-partum, also considering a large set of maternal and infant variables. At the third trimester of pregnancy (T1) and at three months postpartum (T2), 43 primiparous women filled in a set of questionnaires evaluating symptoms of depression (CES-D) or anxiety (STAI), the woman’s parental bonding experiences (PBI), alexithymia (TAS-20), maternal attachment to the baby (PAI, MPAS) and the perceived social support (MSPSS). At T2 mothers also completed a questionnaire on infant temperament and took part in the CARE-Index videotaped procedure. Dyadic sensitivity was predicted by higher maternal trait anxiety scores in pregnancy. In addition, the mother’s experience of being cared for by her father in childhood was predictive of her infant’s lower compulsivity, while paternal overprotection predicted higher unresponsiveness. The results highlight the influence of perinatal maternal psychological well-being and maternal childhood experiences on the quality of the dyadic relationship. The results may be useful to foster mother–child adjustment during the perinatal period.

## 1. Introduction

More than half a century of research has confirmed the central role of the early parent–child relationship for optimal child development in terms of socio-emotional, cognitive and global health outcomes [1,2,3,4,5]. Starting from ego psychology [6,7] and object relations theory [8,9,10,11,12,13,14,15,16], the parent-child relationship has gradually gained more attention in psychoanalytic theory. In this context, attachment theory played an important role in highlighting the developmental function of the relationship between caregiver and infant in childhood. As proposed by Bowlby [17,18,19,20], and further elaborated by more recent attachment theorists [21,22,23,24,25], the attachment relationship can influence psychological functioning and social relationships throughout the life cycle. A child’s secure attachment has a positive impact on his/her later development and well-being; in contrast, an insecure attachment has a negative impact on his/her well-being, as widely demonstrated by research in this field [26,27,28,29]. Thanks to attachment studies, early child–caregiver interaction began to be investigated in experimental settings, paving the way for the advent of infant research which integrated psychoanalytic theory with attachment theory, developmental psychology, neuroscience and systemic theories [30,31,32]. This new approach, through the study of micro-sequences in videotaped infant–caregiver interactions, has made it possible to bring unequivocal evidence of the complexity of these early preverbal exchanges, the infant’s early skills and the infant–caregiver mutual influence that Stern [30] has compared to a “dance”.

Maternal sensitivity [21], defined as the ability to detect and respond appropriately to the child’s needs within his/her zone of proximal development [21,33,34], is the key characteristic of interactions promoting the attachment security of the child [35].

The concept of maternal sensitivity, as proposed by Mary Ainsworth [21], has been repeatedly expanded and redefined by the literature, starting from the previous definition of “primary maternal preoccupation” [16] and ranging from the maternal reflective function as the basis of mentalization [24,36] to mind-mindedness [37] and even further. Overall, maternal sensitivity concerns the capacity to identify with the child and thus recognize his/her needs, representing the child in terms of a subject of mental states. Recent studies underlined the importance of longitudinal studies and observational data regarding the mother’s sensitivity and well-being [38]. 

Becoming a mother is a psychological passage of great complexity characterized by profound changes that affect the entire sphere of the life of the woman. Psychoanalytic literature introduced the term “transition to motherhood” underlining the intensity of the emotional upheavals that characterize the psychological processes of motherhood and make it a phase of “maturation crisis” which aims to redefine the woman’s identity in terms of maternal identity [39,40]. These psychological processes imply a re-elaboration of the bond experiences that the woman lived with her parents in childhood, namely her own parental models. From a psychodynamic point of view, it is believed that, in turn, the quality of experiences lived in the relationships with one’s parents influences the sensitivity of the parent with his/her own child, as well as the structuring of the care-giving system [36,41,42,43]. Maternal sensitivity can be seen as the result of complex processes of re-elaboration of the experiences of care in the significant relationships that the woman experienced in her childhood. Furthermore, the quality of early emotional relationships influences the development of the individual’s emotional regulation, and specific difficulties at this stage may affect the individual’s ability to recognize, represent and process emotions in adult life (alexithymia as defined by Sifneos [44]). 

The particular complexity of the perinatal period also makes it a period of increased psychopathological risk [45]. The literature shows that during the perinatal period, about 11.9% of women suffer from depression [46] and about 15–17% of women suffer from anxiety, 5% of which experience both anxiety and depression [47].

Considering the increased risks to women’s mental health in the perinatal period, existing scientific research aimed to understand the effects of depression and anxiety on the sensitivity of the mother–infant interaction [48,49]. It has been demonstrated that the presence of symptoms of depression or anxiety affects the quality of the early postnatal relationship, which appears perturbed in many ways: maternal withdrawal; low availability; distancing; intrusiveness; ambivalence; covert hostility [48,50,51]; less closeness, warmth and mutual attunement [52,53,54]; less accurate identification of positive emotions and a heightened sensitivity to negative emotions to the infant’s face [55], as well as less involvement in positive enrichment activities with the child [56]. A recent meta-analysis further confirmed the negative (even though small) association between maternal perinatal depression and maternal sensitivity, particularly when considering studies that compared depressed vs. nondepressed mothers/control group, where a moderate effect size emerged [57]. More in general, the quality of parenting was proposed as one of the possible mechanisms of intergenerational transmission of depression [58,59]. A recent meta-analysis seems to confirm this hypothesis, identifying an indirect effect (with a small effect size) of maternal depression on several domains of child functioning (e.g., biological, cognitive/intellectual, emotional/behavioral, social functioning, attachment and general psychopathology), mediated by parenting quality [60]. Emotional difficulties affect the mother’s ability to read her child’s needs and expose the child on a prolonged basis to unrepaired interactive mismatches, with detrimental long-lasting consequences for both mother and child [61,62]. These data seem supported further by recent neuroscientific studies, which suggest that depression [63] and anxiety [64] can impact the mother’s neural response to infant cues, influencing detection, interpretation and reaction to the child’s signals.

This becomes evident in the analysis of videotaped micro-sequences that make up the mother–child interactions as suggested by many studies [65,66]. In this line of research, interactions are viewed in dyadic terms, defining a dyadic sensitivity pattern [33,34] as a global index of the quality of the interaction (in addition to the individual indices referring to the mother and the child). As described by Crittenden [33,34]: “Adult sensitivity in play is any pattern of behavior that pleases the infant and increases the infant’s comfort and attentiveness and reduces its distress and disengagement”.

The quality of early interactions that provide the basis for the development of the infantile self is underpinned by specific parenting skills. These abilities can be examined through specific methods that analyze early interactions in terms of reciprocal exchanges at the level of preverbal behavior and underlying dispositions [33,34]. An interesting new approach, called parental embodied mentalizing ability (PEM, [67,68]), emphasizes the importance of the preverbal aspects of the interaction, highlighting the relevance of the implicit dialogue between the maternal mind and the developing infant mind. The role of these early experiences of intersubjectivity had already been pointed out by Stern [30] who, in line with infant research data, highlights the child’s innate predisposition to affective attunement as well as the importance of early implicit relational exchanges between caregiver and infant, emphasizing their influence on the development of the self. The aim of this study was to evaluate the potential influence of maternal depressive and anxious symptoms on the quality of mother–child interactions, while also considering a large set of maternal and infantile variables with particular regard to the nature of the woman’s internalized parental bond and alexithymia. The internalized representations of bonding can be viewed as the junction of intergenerationality, conveying in preverbal exchanges profound meanings with respect to the experiences lived by the parents in their significant childhood relationships. Parental bonding has been investigated relatively little through self-reports. Therefore, it might be interesting to know if parental reporting of internalized parental bonding can be informative at the level of prevention to foster mother–child adjustment during the perinatal period. A further aim was to compare the maternal rating of child temperament with characteristics of dyadic interaction rated by independent coders to evaluate whether there is an agreement between the two different points of observation of the child’s behavior.

## 2. Materials and Methods

### 2.1. Research Design

The study was a longitudinal survey aimed to assess the relation between maternal perinatal psychological state and dyadic functioning in mother–infant interactions at three months post-partum, while also considering specific maternal variables (parental bonding, alexithymia, pre and postnatal attachment to the baby, perceived social support) and infant temperament.

Alternative hypotheses were:

**Hypothesis 1 (H_1_).**  *Higher levels of perinatal depression, anxiety and alexithymia are negatively associated with measures of dyadic functioning at three months post-partum.*

**Hypothesis 2 (H_2_).**  *Higher levels of care and lower levels of overprotection in maternal experiences of childhood parental bonding are positively associated with measures of dyadic functioning at three months post-partum.*

**Hypothesis 3 (H_3_).**  *Higher levels of temperamental difficulties are negatively associated with measures of dyadic functioning at three months post-partum.*

### 2.2. Participants

The current study is part of a larger project focused on mother and child well-being during pregnancy and postpartum [69]. The research study was proposed to a sample of pregnant women attending the childbirth education class or the third-trimester ultrasound session in four health services of the National Health System in Brescia, a medium-sized northern Italy city. Women were included in the study according to the following criteria: Italian speaking, over 18 years old, primipara, singleton pregnancy, in the third trimester of pregnancy. This study reports data on the subset of 43 women who accepted to be visited at home and participate in the CARE-Index videotaped procedure.

### 2.3. Procedure

Ethical approval was obtained from the health services Ethics Committee, written informed consent was required from the women to participate. A set of questionnaires were administered during the third trimester of pregnancy (T1) and at three months post-partum (T2) to the entire sample who participated in the larger project. The third trimester of pregnancy and the period around three months postpartum were chosen because they represent two moments in which the risk of depression is well documented [70]. Furthermore, at three months postpartum, the mutual regulation of mother–child interaction is already established [71]. At T1 women filled in the questionnaires while attending the perinatal health services; at T2 the questionnaires were sent to their homes in a prepaid envelope. In addition, 43 women also agreed to be videotaped during a play interaction with their three-to-four-month-old babies. The present study reports data on this subsample of women. The videotaped interactions were recorded at home, the mothers were asked to play with their baby as they usually would for 3–5 min. The taped interactions were coded by three reliable coders as recommended by Crittenden [33,34]. The coders were blind to the research hypotheses, and inter-rater reliability was adequate (Cohen’s k = 0.80). As prescribed by the Ethics Committee, women who either exceeded the clinical threshold for anxiety/depression or scored more than 0 to question 10 of the EPDS were called by phone to ensure that they had correctly understood the questions and to talk about the results and their psychological state. An appointment was then proposed with the psychologist of the perinatal clinic in the woman’s area of residence. These women were also sent a letter that they could deliver to their family doctor about the test results.

### 2.4. Measures 

Participants filled in the following questionnaires:

Pregnancy assessment (T1):

A questionnaire gathering and evaluating socio-demographic characteristics, pregnancy-related information and psychosocial risk factors. 

The Center for Epidemiological Studies-Depression Scale (CES-D; [72,73]), measuring the level of depression symptoms in the past week; the clinical cut-off score was ≥16.

The State–Trait Anxiety Inventory Form Y (STAI; [74,75]), consisting of two scales measuring state anxiety (STATE-A) and trait anxiety (TRAIT-A), respectively; the clinical cut-off score was ≥41.

The 20-Toronto Alexithymia Scale (TAS-20; [76,77]), assessing the alexithymia construct; the TAS-20 total score was used as an indicator of the possible presence of alexithymic traits; the clinical cut-off score was ≥51.

The Parental Bonding Instrument (PBI; [78,79]), comprising two scales termed care and overprotection, evaluating parental styles as perceived in childhood. The measure is retrospective, meaning that adults are to complete the measure for how they remember their parents during their first sixteen years. 

The Multidimensional Scale of Perceived Social Support (MSPSS; [80,81]), assessing the social support perceived by the woman from family, friends and significant others. 

The Prenatal Attachment Inventory (PAI; [82,83]), investigating maternal emotional involvement with the unborn child.

Post-partum assessment (T2):

A questionnaire surveying delivery and puerperium characteristics. 

A new administration of CES-D, STAI and MSPSS.

The Edinburgh Postpartum Depression Scale (EPDS; [84,85,86]), assessing post-partum symptoms of depression; the clinical cut-off score was ≥10.

The Maternal Postnatal Attachment Scale (MPAS; [87,88]), evaluating maternal postnatal attachment through three dimensions: quality (MPAS q.), acceptance (MPAS a.) and pleasure in proximity (MPAS p.). 

The Early Infant Temperament Questionnaire (EITQ; [89,90]), evaluating infant temperament on nine subscales reflecting independent dimensions of infant temperament: *activity* (the amount of physical motion during eating, dressing or bathing), rhythmicity (the regularity of physiologic functions such as hunger and sleep), approach/withdrawal (the nature of initial responses to new stimuli), adaptability (the ease with which difficult reactions to stimuli can be modified in a desired way), intensity (the energy level of responses), mood (the amount of pleasant or unpleasant behavior in various situations), persistence (the length of time particular activities are pursued by the child), distractibility (the effectiveness of extraneous environmental stimuli in interfering with ongoing behaviors) and sensory *threshold* (the amount of stimulation, such as sounds or light, necessary to evoke discernible responses in the child). As a measure of global temperament difficulty, the subscales of EITQ were summed to yield a sum score of EITQ. 

Mothers and children also participated in the CARE-Index procedure [33,34], a video-recorded procedure of parent–child interaction providing 8 indicators of the quality of interaction on a 0–14 points scale. The CARE-Index is a dyadic procedure that assesses adult sensitivity in a dyadic context. The CARE-Index provides an overall measure of *dyadic sensitivity*; three maternal scales: sensitivity (ability to understand the baby’s signals and respond to them appropriately), control (subtle tendency to force the child into the mother’s schemes) and unresponsiveness (inability to pick up on the baby’s cues and distancing); four baby scales: cooperation (participation and enjoyment of the interaction), compulsivity (blockage or excessive compliance due to fear of the adult in the interaction), difficulty (the degree of protests and loudness of the child) and passiveness (lack of initiative and poor tone). The dyadic sensitivity is the global index that best describes the quality of mother–child interaction. The quality of mother–child interactions is classified according to the dyadic sensitivity score as follows: at risk (0–4), inept (5–6), adequate (7–10) or sensitive (11–14).

### 2.5. Statistical Analyses 

Descriptive statistics were calculated for quantitative and qualitative variables. Total scores of the questionnaires were analyzed as continuous or dichotomous variables using the appropriate cut-offs. The Cronbach’s α coefficient was used to evaluate the internal consistency of the questionnaires. Maternal psychological well-being was defined as low scores on the depression, anxiety and alexithymia questionnaires. 

The CARE-Index scale scores were dichotomized using quartiles in order to divide the sample into high- and low-quality interactions. Spearman coefficients were used to examine associations between maternal sociodemographic, obstetric and psychosocial characteristics, as well as mother and child variables and the CARE-Index indicators of interaction quality. Finally, binary logistic regression models were used to examine whether any of the mother and child variables acted as exogenous variables predictive of CARE-Index indicators of interaction quality. All data analyses were performed using SPSS (version 22.0) for Windows.

## 3. Results

### 3.1. Descriptive Analysis

A total of 112 mothers (76.7%) filled in the questionnaires at T1 and T2. Five women were excluded from subsequent analysis due to preterm delivery (3.6%) or missing data (0.9%). The sample, therefore, consisted of 107 women, among whom a total of 43 women (40.19%) accepted to be videotaped at home while interacting with their three-to-four-month-old babies. The characteristics of the sample are shown in Table 1. Most of the women in the study sample were married or cohabiting and highly educated; 97% of women had a job but were on maternity leave at the time of assessment (T2). The vast majority had a planned pregnancy, and all the children were born healthy. Differences between accepting women and the remaining sample have been analyzed with regard to all demographic and psychosocial variables of women and children. The analysis showed no statistically significant difference between the two groups. Only the CES-D scores (depression symptoms) showed a trend towards significance, perhaps suggesting that women with higher depression scores were more motivated to meet experimenters at home. The questionnaires administered at T1 and T2 demonstrated adequate psychometric properties (see Table 2 and Table 3). Scores above the clinical cut-off in the questionnaires are shown in Table 2. Symptoms were usually mild or moderate and social support appeared particularly high [81]; only two women showed elevated symptoms on postnatal anxiety and depression questionnaires and a positive score (low) on the suicidal ideation item. Women who fit these criteria were contacted and referred to the local perinatal clinic. 

Descriptive statistics of the questionnaires measuring maternal well-being at T1 and T2 and statistical results of the paired comparison *t*-test are reported in Table 2 and Table 3; CARE-Index scales are reported in Table 4; Table 5 displays the children’s temperament scales. The types of mother–child interactions, classified on the basis of the dyadic sensitivity score, are displayed in Table 6.

### 3.2. Correlational Analysis

Table 7 shows Spearman’s statistical correlations between maternal questionnaire scores, CARE-Index and temperament scales. Higher dyadic sensitivity scores correlated with lower trait-anxiety and lower alexithymia scores in pregnancy. With respect to the maternal scales of the CARE-Index, less unresponsive mothers appeared to have had higher pregnancy depression scores and to have perceived their father as less overprotecting in their childhood. Maternal sensitivity was also positively correlated with child adaptability and intensity. Considering the baby’s scales of CARE-Index, lower maternal alexithymia scores, lower postnatal depression scores, and lower antenatal and postnatal trait anxiety correlated with a higher score in baby’s cooperation. With regard to the woman’s bond with mother and father (PBI), higher maternal care and paternal care scores (PBI) correlated with a lower compulsivity of the child. Higher paternal overprotection and EPDS scores correlated with a lower difficulty of the child. 

Among the sociodemographic, obstetric, and psychosocial variables, only the duration of the couple relationship correlated with dyadic sensitivity (ρ = 0.325, *p* < 0.05). 

### 3.3. Logistic Regression Analysis

According to the significant results emerging in the bivariate analysis, a series of logistic regression analyses were performed on the CARE-Index scales. The significant models are illustrated below: 

A logistic regression was performed to ascertain the effect of the duration of the couple´s relationship, TAS and trait anxiety at T1 on dyadic sensitivity. The logistic regression model was statistically significant χ^2^ (1, N = 43) = 7.830 *p* = 0.005. The model explained 22.3% (Nagelkerke R^2^) of the variance in dyadic sensitivity. A higher level of trait anxiety in pregnancy predicted a lower dyadic sensitivity at three months post-partum (OR = 1.14, 95% CI [1.03, 1.28]).

A logistic regression was performed to ascertain the effect of CES-D during pregnancy and paternal overprotection on maternal unresponsiveness. The logistic regression model was statistically significant χ^2^ (2, N = 43) = 10.067 *p* = 0.007. The model explained 31.7% (Nagelkerke R^2^) of the variance in maternal unresponsiveness. Higher levels of CES-D in pregnancy and lower levels of paternal overprotection predicted lower levels of maternal unresponsiveness at three months post-partum (OR = 0.796, 95% CI [0.66, 0.99]).

A logistic regression was performed to ascertain the effect of paternal and maternal care on child compulsivity. The logistic regression model was statistically significant χ^2^ (2, N = 43) = 9.056 *p* = 0.011. The model explained 27.4% (Nagelkerke R^2^) of the variance in child compulsivity. A higher level of paternal care predicted a lower level of child compulsivity at three months post-partum (OR = 0.883 95% CI [0.78, 0.99]). 

## 4. Discussion

Using a longitudinal design, the present study explored the influence of perinatal psychological symptoms and maternal experiences with her parents in childhood on mother–child interaction in the postpartum.

The first hypothesis of the study concerned the potential influence of different aspects of maternal psychological well-being, defined in terms of low scores of depression, anxiety and alexithymia, on the quality of dyadic functioning in mother–infant interactions at three months after delivery.

According to H_1_, the dyadic sensitivity score, considered as the best indicator of the quality of the mother–child interaction, is inversely correlated with the alexithymia score. In line with the hypotheses, this result confirms that a mother’s ability to cope with emotional states, representing them explicitly/verbally, is associated with a better dyadic adjustment. Dyadic sensitivity was also higher when mothers were in a long-standing relationship, confirming the importance of a consolidated relationship with the partner as a support for a better mother–child adjustment. Furthermore, higher trait anxiety in pregnancy was associated with lower dyadic sensitivity; more importantly, this was the only variable that remained significant in the regression model predicting dyadic sensitivity. These results seem to suggest that women who perceive themselves as anxious may have more difficulties overcoming the maturational crisis inherent in the processes of transition to motherhood [39,40]. This, in turn, can impact the quality of interaction with their infant in the postpartum. 

A singular result showed that higher levels of depression in pregnancy were associated with lower levels of maternal unresponsiveness, partially in contrast with H_1_, while the experience of an overprotecting father was associated with increased maternal unresponsiveness in the postpartum, according to H_2_. These results were confirmed both in the correlational and regression analyses. While the negative relation between maternal depression in pregnancy and unresponsiveness is somewhat surprising, our data seems to suggest an important role of a certain amount of depressive symptoms in the last period of pregnancy as a predictor of better maternal adaptation. Psychoanalytic theory has drawn attention to the profound psychic upheaval which women live through during the transition to motherhood. When this process is gradually resolved, through a possibility of elaboration of the emotions linked to the transformations of motherhood, this resolution allows the foundation of the maternal identity and supports the attainment of the maternal role, as supported by previous studies [91,92]. 

On the other hand, as hypothesized, women’s experiences of an overprotecting father were associated with lower levels of maternal responsiveness. While more studies are needed to confirm our findings, it can be hypothesized that overprotection in the family of origin can negatively affect the process of individuation, influencing the assumption of the maternal identity and role; this is manifested by the lower levels of responsiveness in the interactions with the infant shown in this study. In addition, some authors suggested that experiences of overprotection in infancy can undermine the development of a sense of control, increasing anxiety, and the person’s sense of threat [93,94,95]. This, in turn, can negatively influence women’s representation of themselves as a mother, impacting responsiveness in the interaction with her child. The relation between trait anxiety and dyadic sensitivity found in the current study seems to go in this direction.

Perhaps the most striking and interesting finding of the study, according to H_2_, was the association between the mother’s bond with her parents in childhood and the quality of her current interaction with her infant. Higher scores on the PBI’s maternal care and paternal care scales correlated with less compulsivity in the child, that is, the child feels welcomed and does not perceive his/her own reactions as dangerous for the mother, therefore, he/she behaves freely in the interaction. This interesting result indicates that the maternal experiences of care received from her parents in childhood are associated with the greater well-being of her child in the interaction, emphasizing the intergenerational transmission of affective meanings within the early mother–infant interaction. Furthermore, paternal care remained in the regression as an independent predictor of the child’s lower compulsiveness, highlighting how much a loving and caring father figure is influential in the quality of the current relationship between mother and child.

Partially in contrast with H_3_, dyadic quality indicators were not associated with child temperament, except for child adaptability and intensity which correlated positively with maternal sensitivity. However, these results were found only at the bivariate level, and they were not confirmed in the regression model. This result highlights that there is a discrepancy between the mothers’ reports of their infants’ temperament and the observations of the infant’s behavior in the interaction carried out by independent observers. Inconclusive overlap between different measures of temperament had previously been reported by Woorobey [96]. In our study, maternal sensitivity was correlated with a child who is less adaptable and more intense. This result, which might seem unexpected, can actually highlight sensitivity as a greater maternal capacity to accept the child in his/her manifestations. 

A further unexpected result is that in this small sample of women with low psychosocial risk and high social support, there was a lower-than-average presence (32.6% compared with approximately 50% expected [33,34]) of interactions classified as adequate or sensitive. This percentage, more similar to that of at-risk samples [97,98], can perhaps be explained by sample self-selection: women with greater emotional distress (as seen in the comparison between the global sample and the sample of women who accepted the home visit) could have joined in at a higher percentage, maybe due to their need for support. Nonetheless, the number of women who exceeded the cut-off for symptoms of depression or anxiety was not higher than expected. However, it is possible that there were subthreshold levels of distress or concern in these mothers that influenced the difficulties encountered in interactions that were not captured in our study. It is also possible that in this sample of women in their first motherhood experience, the highlighted difficulties in early interactions are attributable to inexperience and to the natural adjustment process between mother and child that occurs gradually and continues even after three months. It must be considered that a score of five or six in the dyadic sensitivity may indicate a transient difficulty. In this small sample of primiparous mothers, the interactive difficulty in some dyads does not seem to be explained only by the maternal perinatal depression or state anxiety scores. Instead, the role of variables more linked to the woman’s history and personality seem to be highlighted, such as alexithymia, trait anxiety and experiences of bonding with parents in the woman’s childhood.

## 5. Limitations of the Study

The limits of the study are represented by the small size of the sample and by the characteristics of the women (high level of education, employed, high social support, low psychosocial and obstetric risk and married or cohabiting). A further limitation is represented by the use of self-report questionnaires to assess depressive or anxious symptoms instead of diagnostic interviews, and the absence of an assessment of paternal psychological well-being. Furthermore, no specific tools were used to detect maternal concerns in the postpartum period [99], which might be more appropriate than tools for general anxiety and depression. Future studies are needed to investigate dyadic sensitivity in larger samples, samples at psychosocial or obstetric risk, foreign mothers, same-sex couples or single mothers. The role of paternal psychological well-being needs to be studied further as well.

## 6. Conclusions

Through a longitudinal perspective, the study highlighted the influence of maternal prenatal trait-anxiety symptoms on mother–infant interactions, underlining the importance of screening for perinatal psychological difficulties and supporting mothers throughout their transition to motherhood [100,101,102]. Perhaps the most interesting result of the study was the influence of the parental caring relationships experienced by the woman in her childhood with respect to the characteristics of the current mother–child interactive exchanges. Internalized positive maternal experiences of care seem to recur in early interactive modalities, evidenced in a less compulsive style of the child in the interaction. On the other hand, perceived paternal overprotection in childhood was related to higher levels of maternal unresponsiveness. Notably, these results were found by independent observers who were blinded to the hypotheses of the study. 

The data underline the importance of taking care of the woman in the perinatal period not only considering her psychological well-being but also her family history and her own experiences in caring relationships with her caregivers. As proposed by Cramer [103] early interaction aspects of parental history are re-enacted, constituting the central nucleus of intergenerationality; however, they are also a point from which a change can be fostered in the parent [104].

## Figures and Tables

**Table 1:** Demographic, perinatal and psychosocial characteristics of the women and the children of the sample (N = 43).

Women	Mean (SD)
Age	31.81 (3.33)
Years of relationship with the partner	7.75 (4.12)
Primiparous	43 (100%)
**Education**	**Frequencies (%)**
High school/University	35 (81.4%)
** Employment status **	**Frequencies (%)**
Office employee	19 (44.2%)
Professional	6 (14%)
Factory worker	4 (9.3%)
Technical graduate	2 (4.7%)
Teacher	2 (4.7%)
Other	10 (23.1%)
Unemployed	1 (2.3%)
**Marital status**	**Frequencies (%)**
Married	33 (76.7%)
** Pregnancy, delivery, and breastfeeding **	**Frequencies (%)**
Planned pregnancy	37 (88.1%)
Attended childbirth course	43 (100%)
Recruited during the childbirth course	39 (92.7%)
Previous miscarriages	5 (11.6%)
Delivery complications	12 (27.9%)
Breastfeeding at three months post-partum	31 (72.1%)
**Stressful events**	**Frequencies (%)**
One or more event in the past year	25 (58.1%)
**Psychiatric history**	**Frequencies (%)**
History of anxiety or depression symptoms	8 (18.6%)
Currently on psychiatric drugs	0 (0%)
Previously on psychiatric drugs	1 (2.3%)
Previous psychotherapy	2 (4.6%)
**Children**	**Mean (SD)**
Age (in days)	95.37 (5.30)
Weight (in grams)	3320.81 (389.98)
**Children’s gender**	** Frequencies (%) **
Male	48.8%
Female	51.2%

**Table 2:** Descriptive statistics of the questionnaires administered to the women at T1 and T2 (N = 43) and the number of women above the clinical cut-off.

Questionnaires	T1 Mean (SD)	T1 α	T1 Cut-Off N > (%)	T2 Mean (SD)	T2 α	T2 Cut-Off N > (%)	*t*
CES-D	9.70 (5.68)	0.76	6 (14%)	6.67 (9.64)	0.79	4 (9.3%)	1.98
STATE-A	32.72 (6.53)	0.89	7 (16.3%)	31.23 (9.43)	0.90	3 (7.0%)	1.39
TRAIT_A	35.26 (7.76)	0.90	8 (18.6%)	33.30 (8.88)	0.91	5 (11.6%)	1.34
MSPSS	6.05 (.77)	0.91		5.96 (1.05)	0.90		1.08

CES-D = Center for Epidemiological Studies–Depression; STATE-A = State Scale of STAI; Trait-A = Trait Scale of STAI; MSPSS = Multidimensional Scale of Perceived Social Support; α = Cronbach’s Alpha; *t* = *t*-test.

**Table 3:** Descriptive statistics of the questionnaires administered only at T1 or at T2 (N = 43).

Scales T1	Mean (SD)	Cronbach’s α
PAI	63.35 (8.33)	0.84
TAS-20	41.72 (11.44)	0.80
CARE-M	26.00 (7.12)	0.88
OVPR-M	15.09 (8.14)	0.86
CARE-P	22.51 (7.60)	0.87
OVPR-P	14.83 (9.00)	0.88
Scales T2	Mean (SD)	Cronbach’s α
EPDS	4.65 (5.35)	0.83
MPAS	81.74 (8.06)	0.78
MPASq	40.16 (3.55)	0.63
MPASa	20.72 (2.54)	0.58
MPASp	16.30 (2.39)	0.60

PAI = Prenatal Attachment Inventory; TAS-20 = Twenty Toronto Alexithymia Scale; PBI = Parental Bonding Instrument; CARE-M = Maternal Care dimension of PBI; CARE-P = Paternal Care dimension of PBI; OVPR-M = Maternal Overprotection dimension of PBI; OVPR-P = Paternal Overprotection dimension of PBI; MPAS = Maternal Postnatal Attachment Scale; MPASq = quality dimension; MPASa = acceptance dimension; MPASp = pleasure in proximity dimension; EPDS = Edinburgh Postpartum Depression Scale.

**Table 4:** Descriptive statistics of CARE-Index scales (N = 43).

CARE-Index Scales	Mean (SD)	Range
Dyadic sensitivity	5.77 (2.64)	2–13
Maternal sensitivity	5.72 (2.60)	2–13
Maternal control	4.60 (3.51)	0–11
Maternal unresponsiveness	3.67 (3.46)	0–12
Baby cooperant	5.58 (2.91)	1–13
Baby compulsive	2.07 (2.54)	0–12
Baby difficult	4.07 (3.00)	0–11
Baby passive	2.28 (3.16)	0–12

**Table 5:** Descriptive statistics of EITQ subscales of temperament (N = 43).

EITQ Subscales	Mean (SD)	Cronbach’s Alpha
Activity (high)	3.51 (0.74)	0.60
Rhythmicity (irregular)	3.30 (0.71)	0.67
Approach (withdrawing)	2.67 (0.80)	0.55
Adaptability (low)	2.55 (0.75)	0.47
Intensity (intense)	3.67 (0.90)	0.56
Mood (negative)	2.90 (0.71)	0.72
Persistence (low)	2.34 (0.66)	0.57
Distractibility (low)	2.40 (0.76)	0.36
Threshold (low)	4.01 (0.68)	0.58
EITQ sum (difficulty)	27.36 (3.30)	0.76

Note: Higher numerical scores denote the characteristics in parentheses.

**Table 6:** Frequencies and percentages of mother–infant interaction categories ranked according to the dyadic sensitivity score (N = 43).

Risk Status	F (%)
Sensitive (11–14)	2 (4.7%)
Adequate (7–10)	12 (27.9%)
Inept (5–6)	10 (23.2%)
At risk (0–4)	19 (44.2%)

**Table 7:** Spearman’s correlations between CARE-Index scales, maternal well-being scales, and temperament scales.

	Dyadic Sensitivity	Sensitivity	Control	Unresponsiveness	Cooperation	Compulsivity	Difficulty	Passiveness
Antenatal CES-D	−0.034	0.201	0.186	−0.338 *	−0.051	0.036	−0.080	−0.106
Postnatal CES-D	−0.290	−0.153	0.191	0.004	−0.369 *	0.057	−0.122	0.155
Antenatal STATE-A	−0.104	0.060	−0.147	0.086	−0.154	−0.028	−0.116	0.008
Postnatal STATE-A	−0.030	0.042	0.150	−0.148	−0.112	0.173	−0.281	0.023
Antenatal TRAIT-A	−0.440 **	−0.092	−0.028	0.065	−0.439 **	−0.014	0.049	0.041
Postnatal TRAIT-A	−0.246	−0.054	0.043	−0.006	−0.328 *	0.142	−0.084	−0.022
Antenatal MSPSS	0.051	−0.018	−0.103	0.241	0.067	−0.245	−0.120	0.160
Postnatal MSPSS	0.064	−0.014	−0.132	0.151	0.097	−0.168	0.091	0.043
EPDS	−0.062	−0.004	−0.040	0.034	−0.110	0.054	−0.312 *	0.163
Maternal Care (PBI)	0.176	0.188	−0.019	−0.147	0.131	−0.317 *	0.299	−0.236
Maternal Overprotection (PBI)	0.042	0.160	−0.100	0.021	0.063	0.100	−0.175	0.011
Paternal Care (PBI)	0.249	0.159	−0.204	−0.061	0.173	−0.416 **	0.017	0.111
Paternal Overprotection (PBI)	0.107	0.133	−0.306	0.326 *	0.148	0.033	−0.388 *	0.204
TAS	−0.304 *	−0.206	0.090	0.107	−0.375 *	0.045	0.236	0.049
MPAS	0.242	0.200	−0.090	−0.082	0.265	−0.240	0.019	−0.019
Activity (high)	−0.011	0.254	0.092	−0.216	−0.019	0.077	−0.048	−0.111
Rhythmicity (irregular)	−0.096	−0.024	−0.034	0.084	−0.116	0.032	0.103	−0.041
Approach (withdrawing)	0.219	0.184	0.008	−0.153	0.257	−0.024	0.118	−0.247
Adaptability (low)	0.025	0.322 *	−0.079	−0.096	−0.002	0.026	−0.006	−0.097
Intensity (intense)	−0.026	0.321 *	−0.053	−0.253	−0.065	0.014	0.147	−0.184
Mood (negative)	−0.204	0.074	0.084	−0.067	−0.238	0.065	0.017	−0.051
Persistence (low)	−0.180	−0.235	0.280	−0.075	−0.187	0.034	0.105	0.011
Distractibility (low)	−0.089	0.150	−0.068	−0.046	−0.165	−0.004	−0.177	0.214
Threshold (low)	0.043	0.174	0.107	−0.199	−0.002	0.020	0.040	−0.168
EITQ sum (difficulty)	−0.042	0.256	0.084	−0.230	−0.087	0.069	0.080	−0.187

* *p* < 0.05; ** *p* < 0.01. Note: for dyadic sensitivity, sensitivity, control, unresponsiveness, cooperation, compulsivity, difficulty, and passiveness: 0 = low; 1 = high.

## Data Availability

The data that support the findings of this study are available on request from the corresponding author.
